# Effectiveness and safety of tocilizumab in refractory noninfectious uveitis: a systematic review and meta-analysis

**DOI:** 10.3389/fphar.2025.1694311

**Published:** 2025-12-04

**Authors:** Huanmin Kang, Xue Wu, Hanyue Xu, Yufan Huang, Ming Zhang

**Affiliations:** Department of Ophthalmology, West China Hospital, Sichuan University, Chengdu, Sichuan, China

**Keywords:** tocilizumab, uveitis, interleukin-6 inhibition, systemic autoimmune disease, macular edema, visual acuity, corticosteroid-sparing

## Abstract

**Purpose:**

The aim of this study was to comprehensively evaluate the efficacy and safety of tocilizumab in patients with refractory noninfectious uveitis (NIU) through a systematic review and meta-analysis.

**Methods:**

A comprehensive literature search was conducted across PubMed, Embase, Web of Science, Cochrane Library, and ClinicalTrials.gov from inception to 13 July 2025. Eligible studies included case series and cohort studies evaluating clinical outcomes of tocilizumab in NIU. Study quality was assessed using the Joanna Briggs Institute Case Series Checklist and Newcastle–Ottawa Scale. A single-arm meta-analysis was performed using a random-effects model.

**Results:**

Thirteen studies involving 374 patients were included. The pooled rate of sustained inactive uveitis was 57.08% (95% CI: 46.94%–66.96%), and the overall inflammation remission rate at the final follow-up was 75.23% (95% CI: 64.04%–85.09%). Macular edema resolved in 93.22% of patients (95% CI: 86.76%–98.01%), with a mean reduction in a central macular thickness of 143.57 µm. Mean visual acuity improved by −0.29 logarithm of the minimum angle of resolution (logMAR) (95% CI: −0.55 to −0.04). The pooled glucocorticoid discontinuation rate was 40.25% (95% CI: 13.43%–70.27%). During the follow-up period, the pooled incidence of adverse events and serious adverse events was 13.05% (95% CI: 8.88%–17.78%) and 4.41% (95% CI: 1.08%–9.16%), respectively. Subgroup analysis suggested greater efficacy among patients treated for ≥9 months.

**Conclusion:**

Tocilizumab provides meaningful clinical benefit with acceptable safety in refractory NIU related to autoimmune and inflammatory diseases, particularly in patients with macular edema. These results support the use of tocilizumab as a viable second-line therapeutic option following the failure of conventional immunosuppressants and antitumor necrosis factor alpha agents.

**Systematic Review Registration:**

https://www.crd.york.ac.uk/PROSPERO/view/CRD420251069626, identifier CRD420251069626.

## 1 Introduction

Uveitis is a heterogeneous group of intraocular inflammatory disorders affecting the uveal tract and adjacent ocular structures, including the retina, vitreous, and lens (Chang et al., 2021). It is anatomically classified as anterior, intermediate, posterior, or panuveitis, depending on the primary site of inflammation (Maghsoudlou et al., 2025). With an incidence rate exceeding 50 cases per 100,000 person-years, uveitis remains a major cause of visual impairment and legal blindness worldwide (García-Aparicio et al., 2021). The etiology is diverse, encompassing more than 100 distinct conditions, and is broadly categorized into infectious and noninfectious origins (Tsirouki et al., 2018). Among these, noninfectious uveitis (NIU) accounts for a substantial proportion (20%–94%) and contributes to an estimated 10%–15% of global blindness (Gao et al., 2017; Ramanan, 2025). NIU is generally considered immune-mediated and can be further categorized into cases associated with systemic immune diseases and those limited to the eye (Burkholder and Jabs, 2021). Epidemiological evidence indicates that systemic immune-mediated diseases account for roughly 30%–80% of NIU, most commonly associated with juvenile idiopathic arthritis (JIA), Behçet’s disease (BD), sarcoidosis, and ankylosing spondylitis (Barisani-Asenbauer et al., 2012; Hysa et al., 2021; Joltikov and Lobo-Chan, 2021).

Despite the availability of corticosteroids and conventional immunosuppressants, a significant proportion of patients exhibit refractory disease, characterized by persistent or recurrent inflammation, corticosteroid dependence, and vision-threatening complications such as cataract, glaucoma, and macular edema (ME) (Mehta and Emami-Naeini, 2022). In recent years, biologic agents, particularly antitumor necrosis factor alpha (TNF-α) therapies, have revolutionized the management landscape for NIU (Leone et al., 2023). However, approximately 50% of patients show limited response or intolerance to TNF-α inhibitors, highlighting the need for alternative immunomodulatory strategies (Mesquida et al., 2017).

Interleukin-6 (IL-6) has emerged as a critical cytokine in the pathogenesis of NIU, promoting T-cell activation, upregulating vascular endothelial growth factor (VEGF), increasing vascular permeability, and disrupting the blood–retinal barrier (Mesquida et al., 2017; Mesquida et al., 2019). Elevated intraocular IL-6 levels have been reported under various inflammatory and edematous ocular conditions, including chronic uveitis, diabetic ME, and retinal vein occlusion (Noma et al., 2009; Lin, 2015; Mesquida et al., 2017; Chen et al., 2023). Beyond ocular diseases, IL-6 dysregulation plays a key role in numerous systemic autoimmune and inflammatory diseases. The clinical efficacy of IL-6 inhibition in JIA, rheumatoid arthritis, giant cell arteritis, and Castleman disease supports its pathogenic significance and therapeutic potential (Jones and Jenkins, 2018). The therapeutic success of IL-6 blockade in these systemic disorders has prompted growing interest in its application for NIU, particularly cases associated with systemic immune dysregulation (Yao et al., 2014).

Tocilizumab, a humanized monoclonal antibody targeting the IL-6 receptor, has demonstrated efficacy across various autoimmune and inflammatory diseases, particularly systemic autoimmune disorders such as JIA and rheumatoid arthritis (Sheppard et al., 2017). Several of these systemic autoimmune diseases represent major causes of refractory NIU and are frequently associated with severe, vision-threatening ocular inflammation (El Jammal et al., 2021). Notably, tocilizumab has also shown promising results in managing uveitis secondary to these autoimmune and inflammatory diseases (Atienza-Mateo et al., 2018; Ramanan et al., 2020a; Iannone et al., 2023). For example, Yacine et al. observed a 75% resolution rate of ME and a 67% complete ocular response in patients with anti-TNF-α refractory BD-associated uveitis treated with tocilizumab (Khitri et al., 2023). Conversely, the APTITUDE trial enrolled 21 pediatric patients with anti-TNF-α refractory JIA-associated uveitis and found that only 33% met the resolution of ocular inflammation by week 12 after subcutaneous tocilizumab treatment (Ramanan et al., 2020a). Although these studies suggest potential therapeutic benefit, their limited sample sizes, single-arm designs, and heterogeneous outcome measures constrain the robustness and generalizability of their findings.

Given these limitations, a comprehensive synthesis of current evidence is needed. In this study, we conducted a systematic review and meta-analysis to evaluate the efficacy and safety of tocilizumab in refractory NIU associated with autoimmune and inflammatory diseases. Key outcomes included inflammation remission, resolution of ME, improvement in visual acuity (VA), and glucocorticoid-sparing effects. Subgroup analyses were further performed to assess the influence of treatment duration, patient age, underlying disease, and follow-up length on clinical outcomes.

## 2 Methods

### 2.1 Study design and registration

This systematic review and meta-analysis was conducted in accordance with the Preferred Reporting Items for Systematic Reviews and Meta-Analyses (PRISMA) 2020 guidelines (Page et al., 2021) and was prospectively registered in PROSPERO (CRD420251069626).

### 2.2 Search strategy

We systematically searched PubMed, Embase, Web of Science, the Cochrane Library, and ClinicalTrials.gov from inception to 13 July 2025. The PubMed strategy combined Medical Subject Headings (MeSH) and free-text terms for uveitis and interleukin-6 inhibitors, for example, (“Uveitis” [Mesh] OR uveitides) AND (“Interleukin-6 Inhibitors” [Mesh] OR Inhibitors, Interleukin-6 OR Anti-IL-6 Agents OR Anti-Interleukin-6 Agents OR Tocilizumab). Full search strategies for all databases are provided in Supplementary Table S1. Vocabulary and syntax were adapted for each database.

### 2.3 Eligibility criteria

We used the PICOS framework to guide our eligibility criteria. The inclusion criteria were as follows: (1) population: patients diagnosed with NIU according to the Standardization of Uveitis Nomenclature (SUN) Working Group criteria. Refractory cases were defined as relapse within 3 months after corticosteroid tapering and failure of at least one conventional immunosuppressant and one biologic agent. No restrictions were placed on age or sex; (2) intervention: intravenous or subcutaneous tocilizumab; (3) comparator: not required; single-arm studies were eligible; (4) outcomes: at least one of the predefined efficacy or safety endpoints detailed below in the “Outcome measures”; (5) study design: prospective or retrospective case series or cohort studies enrolling ≥10 participants with ≥6 months of follow-up.

The exclusion criteria were defined as follows: (1) studies that did not explicitly use the SUN criteria to assess intraocular inflammation activity; (2) case reports or small case series (<10 patients), reviews, guidelines, and animal or *in vitro* studies; (3) duplicate publications. When multiple reports described the same cohort, we retained the report with the largest sample.

### 2.4 Outcome measures

The primary outcomes were as follows: (1) sustained inactive uveitis remission, defined as the complete absence of intraocular inflammation for at least 3 consecutive months; (2) ocular inflammation resolution, defined as either a ≥2-step decrease or reduction to grade 0 in anterior chamber cells or vitreous haze according to the SUN criteria.

The secondary outcomes were as follows: (1) ME resolution, defined as a central macular thickness (CMT) < 300 µm with no cystoid space. The absolute change in CMT (µm) from baseline to the last follow-up was also recorded. (2) VA improvement, reported as the mean change in logarithm of the minimum angle of resolution (logMAR) units. The decimal VA was converted to logMAR. (3) Glucocorticoid-sparing effect, captured either as tapering (≥50% dose reduction or ≤5 mg/day prednisone-equivalent) or complete discontinuation. (4) Safety outcomes, recorded as the incidence of adverse events (AEs) and serious adverse events (SAEs). The definition of AEs and SAEs were extracted as reported in the original studies. When not explicitly defined, AEs were defined as any unfavorable or unintended medical occurrence temporally associated with tocilizumab treatment. SAEs were defined as events that were life-threatening, resulted in death, required hospitalization or prolongation of hospitalization, or led to significant disability, consistent with the ICH E2A guideline (International Council for Harmonisation, 1994).

### 2.5 Data extraction

Two reviewers (HM.K and HY.X) independently screened titles/abstracts and full texts. Disagreements were resolved by a third reviewer (M.Z). Data were extracted into a piloted Excel sheet including study characteristics, baseline demographics, prior therapies, tocilizumab regimen, follow-up duration, and all outcomes. When means ± standard deviations were not reported, medians and ranges or interquartile ranges were converted using the methods described by Wan et al. and Luo et al. (Hozo et al., 2005; Luo et al., 2018). If numerical data were presented only in a graphical form, values were extracted using GetData Graph Digitizer (v2.20, getdata-graph-digitizer.com). When necessary data were unavailable, corresponding authors were contacted via email up to three times to obtain missing information. Studies lacking necessary data after these steps were excluded from quantitative synthesis.

### 2.6 Quality assessment

Two reviewers (HM.K and HY.X) independently assessed the risk of bias. Disagreements were resolved by discussion with a third reviewer (M.Z). Case series were evaluated using the Joanna Briggs Institute (JBI) Case Series Checklist (10 items). Studies scoring ≥8 were classified as high quality, 5–7 as moderate quality, and ≤4 as low quality. Cohort studies were assessed using the Newcastle–Ottawa Scale (NOS), which assigns a maximum of 9 stars across three domains: selection, comparability, and outcome. An overall NOS score of 8–9 indicated high quality, scores in the range of 6–7 were considered moderate quality, and scores ≤5 were considered low quality (Luchini et al., 2021; Hilton, 2024).

### 2.7 Statistical analysis

All analyses were performed using Stata 16.0. (StataCorp., College Station, TX, USA). A random-effects model (DerSimonian–Laird method) was applied to generate pooled effect sizes for proportions and weighted mean differences for continuous outcomes, each with a 95% confidence interval. Between-study heterogeneity was evaluated using the Cochran Q test (p ≤ 0.05) and quantified using the I^2^ statistic. Heterogeneity was classified as low (I^2^ ≤ 25%), moderate (25% < I^2^ ≤ 50%), substantial (50% < I^2^ ≤ 75%), and considerable (I^2^ > 75%). If heterogeneity exceeded 25%, potential sources were investigated through prespecified subgroup analyses and sensitivity analyses (leave-one-out method). Publication bias was assessed using Egger’s regression test, with a p-value ≤0.05 indicating significant small-study effects.

## 3 Results

### 3.1 Study selection

**FIGURE 1:**
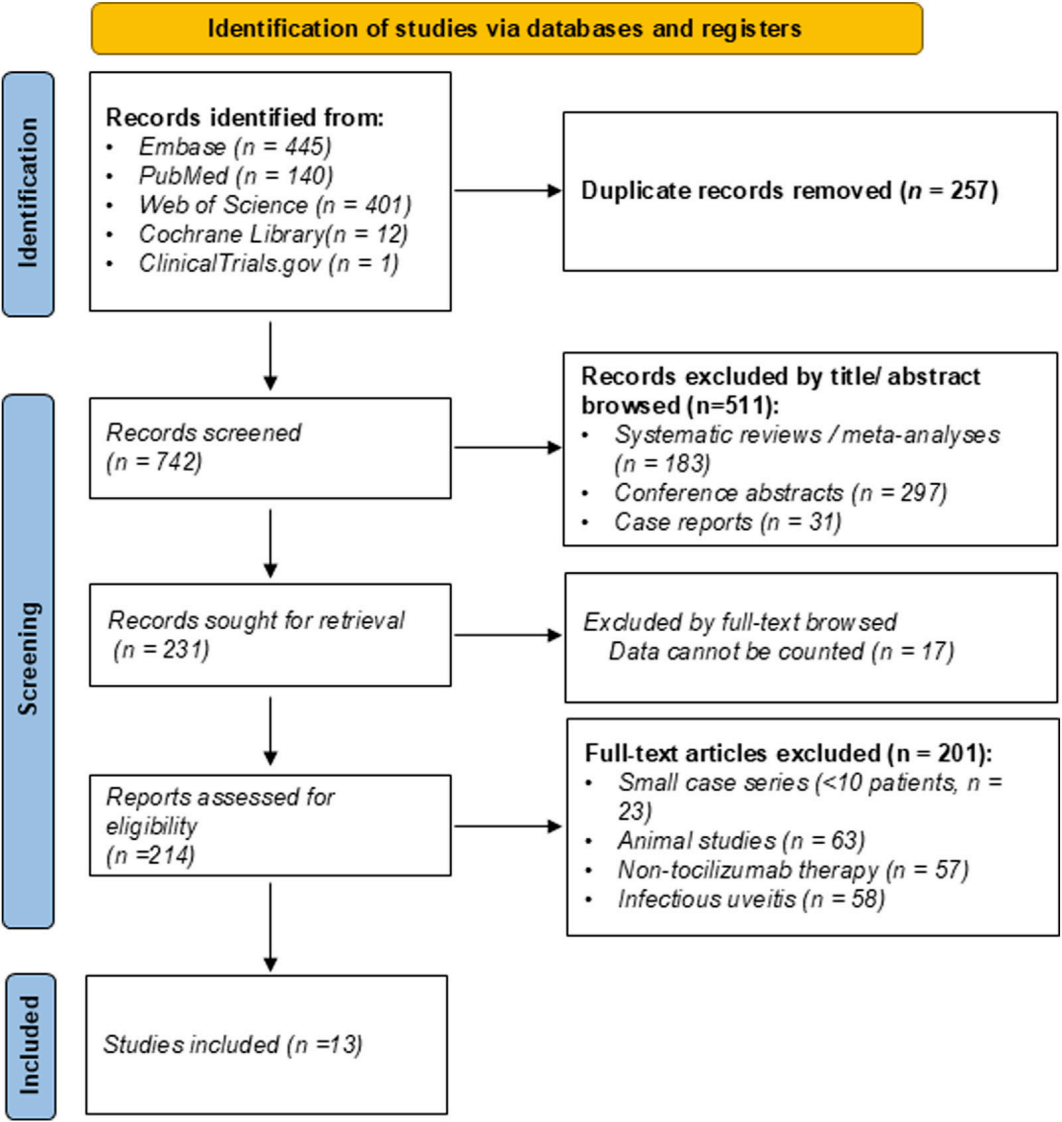
Preferred Reporting Items for Systematic Reviews and Meta-Analyses (PRISMA) 2020 flow diagram of study selection for the systematic review and meta-analysis.

A total of 999 records were identified from Embase (n = 445), PubMed (n = 140), Web of Science (n = 401), Cochrane Library (n = 12), and ClinicalTrials.gov (n = 1). After removing 257 duplicate records, 742 articles were screened by title and abstract. Following a rigorous selection process based on predefined eligibility criteria, 13 studies were included in the final meta-analysis. Two studies initially appeared eligible based on title and abstract screening; however, full-text review revealed that they did not meet the definition of refractory NIU and were therefore excluded (Sepah et al., 2017; Heissigerová et al., 2019). The study selection process is illustrated in the PRISMA flow diagram (Figure 1). Of the included studies, eleven were retrospective single-arm case series, and two were comparative multicenter cohorts: one comparing intravenous vs. subcutaneous tocilizumab, and another comparing tocilizumab vs. anti-TNF agents in refractory NIU (Leclercq et al., 2022; Leclercq et al., 2025).

### 3.2 Study baseline characteristics

Table 1 presents the comprehensive details of the included 13 studies. The 13 studies enrolled 374 patients (131 male and 243 female), predominantly from European centers (12/13) and one from the United States. The pooled mean age was 37.9 years, with three pediatric patient cohorts (mean age ≤16 years) and a maximum mean of 52.0 years. JIA and BD accounted for 47.6% of etiologies; panuveitis was the most frequent anatomical subtype (36.9%). Overall, 78.1% of NIU cases were associated with underlying autoimmune systemic diseases, whereas 82 patients (21.9%) across five studies had ocular-limited NIU. Most patients had received prior biologic immunomodulatory therapy, primarily anti-TNF agents such as adalimumab and infliximab. Conventional immunosuppressants were commonly used before biologic therapy in clinical practice (Beltrán Catalán et al., 2023) but were not consistently specified in the included studies and thus not detailed in Table 1. Tocilizumab was administered predominantly intravenously at 8 mg/kg every 2 or 4 weeks, with occasional subcutaneous dosing (162 mg or 2.9 mg/kg every 1–3 weeks). The median follow-up duration across studies was 30.7 months.

**TABLE 1:** Baseline characteristics of the studies included in the meta-analysis.

Study (first author, year)	Country	Study design	Sample size, n (patients/eyes)	Sex, M/F	Age, mean ± SD, year	Underlying disease (n)	Uveitis type (n)	Prior biologic immunomodulators (n*)	Tocilizumab regimen (n)	Follow-up duration, mean ± SD, month
Tappeiner et al. (2016)	Switzerland	MC-RT, CS	17/30	3/14	15.3 ± 6.9	JIA (17)	Anterior (17)	IFX (13), ADA (7), ETN (2), RTX (1), and GOL (7)	IV: 8 mg/kg q4 w (17)	8.0 ± 2.5
Silpa-archa et al. (2016)	United States	SC-RT, CS	10/18	1/9	34.1 ± 13.4	JIA (5), SAR (1), LN (1), and undifferentiated (3)	Anterior (4), intermediate (1), posterior (1), and pan (4)	IFX (8), ADA (3), RTX (1), DAC (1), IVIG (2), and IFN (1)	IV: 4 mg/kg q4w (2), 8 mg/kg q2w (1), and 8 mg/kg q4w (7)	12.6 ± 10.0
Calvo-Río et al. (2017)	Spain	MC-RT, CS	25/47	4/21	18.5 ± 8.3	JIA (25)	Anterior (17), intermediate (2), posterior (2), and pan (4)	ADA (24), ETN (8), IFX (7), ABA (6), ANA (1), GOL (1), and RTX (2)	IV: 8 mg/kg q4w (21), 8 mg/kg q2w (2), and 8 mg/kg q8w (1) SC: 2.9 mg/kg q1w (1)	13.6 ± 4.6
Mesquida et al. (2018)	Spain	SC-RT, CS	12/16	2/10	34.6 ± 15.6	JIA (6), BRC (2), IDIO (2), SO (1), and AS (1)	NR	ADA (10), IFX (5), RTX (3), ABA (2), GOL (2), ETA (1), and ANA (1)	IV 8 mg/kg q4w (12)	31.3 ± 7.7
Atienza-Mateo et al. (2018)	Spain	MC-RT, CS	11/20	7/4	38.5 ± 20.4	BD (11)	Anterior (2), posterior (1), and pan (8)	ADA (8), IFX (4), CAN (1), and GOL (3)	IV: 8 mg/kg q4w (10) SC: 162 mg q1w (1)	9.5 ± 8.1
Vegas-Revenga et al. (2019)	Spain	MC-RT, CS	25/49	8/17	33.6 ± 18.9	JIA (9), BD (7), BRC (4), IDIO (4), and SAR (1)	Anterior (7), intermediate (4), posterior (5), and pan (9)	ADA (19), ETN (2), IFX (8), ABA (3), RTX (2), GLM (2), DAC (1), and ANK (1)	IV: 8 mg/kg q4w (23) and 8 mg/kg q2w (1) SC: 162 mg q1w (1)	12.7 ± 8.3
Ramanan et al. (2020b)	United Kingdom	MC-SA-P2, CS	21/29	3/18	12.3 ± 3.5	JIA (21)	NR	ADA (21)	SC: 162 mg q2w (15) and 162 mg q3w (6)	9
Atienza-Mateo et al. (2021)	Spain	MC-RT, CS	16/28	10/6	36.5 ± 18.2	BD (16)	Anterior (3), posterior (2), and pan (11)	ADA (10), IFX (7), GOL (3), CAN (1), CZP (1), and ETN (1)	IV: 8 mg/kg q4w (13) SC: 162 mg q1w (3)	23.7 ± 10.2
Leclercq et al. (2022)	France	MC-RT, Coh	55/NR	20/35	47.5 ± 7.0	IDIO (34), BD (3), BRC (6), SAR (1), JIA (2), VKH (2), AS (1), and others (4)	Anterior (2), intermediate (8), posterior (19), and pan (26)	Not clearly counted	IV: 8 mg/kg q4w (39) SC: 162 mg q1w (16)	81.4 ± 21.2
Marino et al. (2023)	Italy	SC-RT, CS	13/23	4/9	21.5 ± 7.5	JIA (13)	Anterior (8) and pan (5)	ADA (12), IFX (7), RTX (2), and other TNF-α blockers (10)	SC: 162 mg q1w (8) and 162 mg q2w (5)	30.5 ± 21.6
Khitri et al. (2023)	France	MC-RT, CS	18/NR	7/11	29.4 ± 11.7	BD (18)	NR	ADA (12), IFX (9), INF-α (5), and ANK (3)	IV: 8 mg/kg q4w SC: 2.9 mg/kg q1w	35.0 ± 9.6
Sota et al. (2025)	Italy	MC-RT, CS	15/29	5/10	6.2 ± 2.8	JIA (7), BD (3), mixed connective tissue disease (1), and IDIO (4)	Intermediate (1), posterior (1), and pan (6)	Not clearly counted	IV: 8–12 mg/kg q4w SC: 162 mg q1w	36
Leclercq et al. (2025)	France	MC-RT, Coh	136/NR	57/79	52.0 ± 4.6	BRC (23), SAR (19), BD (15), VKH (2), MFC (2), undifferentiated (62), and others (13)	Intermediate (13), posterior (57), pan (65), and NR (1)	TNF-α blockers (89)	IV: 8 mg/kg q4w (66) SC: 162 mg q1w (70)	25.5 ± 5.8

M, male; F, female; SD, standard deviation; MC-RT, multicenter, retrospective study; SC-RT, single center, retrospective study; CS, case series; Coh = cohort study; MC-SA-P2, multicenter, single-arm, phase 2 trial; ABA, abatacept; ADA, adalimumab; ANK, anakinra; CAN, canakinumab; CZP, certolizumab pegol; DAC, daclizumab; ETN, etanercept; GOL, golimumab; IFN-α, interferon alpha; IFX, infliximab; IVIG, intravenous immunoglobulin; RTX, rituximab; JIA, juvenile idiopathic arthritis; BD, Behçet’s disease; BRC, birdshot retinochoroidopathy; IDIO, idiopathic uveitis; SAR, sarcoidosis; SO, sympathetic ophthalmia; AS, ankylosing spondylitis; VKH, Vogt–Koyanagi–Harada; LN, lupus nephritis; MFC, multifocal choroiditis; IV, intravenous; SC, subcutaneous; NR, not reported. n*, number of medication exposures, not patient count.

### 3.3 Quality assessment

Supplementary Tables S2, 3 summarize the risk-of-bias assessment. Overall, the included studies demonstrated moderate-to-high methodological quality. In particular, all 11 single-arm case series scored ≥8 out of 10 on the JBI Critical Appraisal Checklist. Commonly unmet items were Q7 (incomplete reporting of participants’ clinical information) and Q8 (absence of clear outcomes or follow-up results).

For the two comparative cohort studies, NOS scores were 7 and 8 out of 9. Both studies failed to adequately describe the follow-up adequacy, which is reflected by 0 scores in the “adequacy of follow-up” domain. Additionally, one study received only one star in the “assessment of comparability” item due to unmatched baseline characteristics.

### 3.4 Meta-analysis

#### 3.4.1 Sustained inactive uveitis remission

**FIGURE 2:**
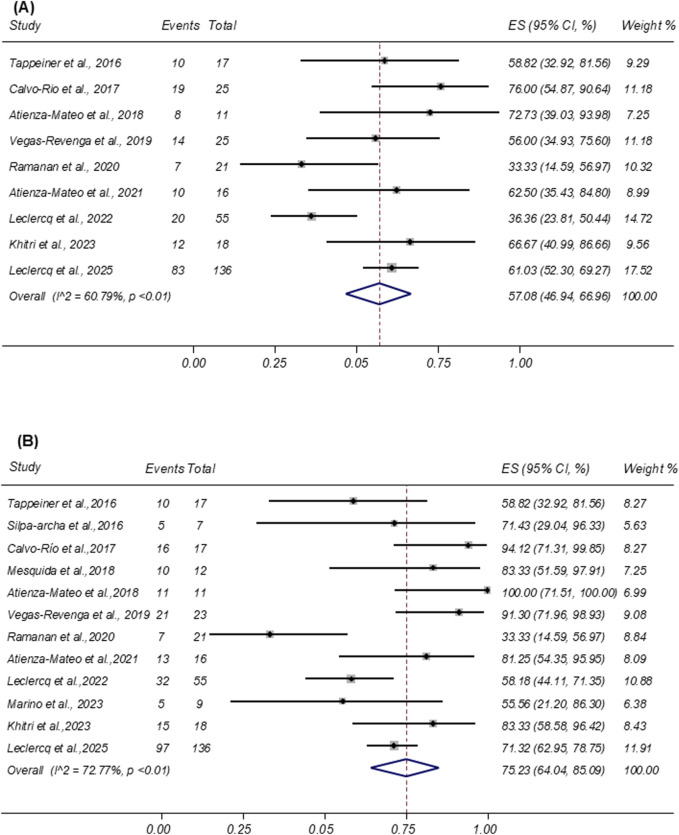
**(A)** Forest plot of studies reporting the rate of sustained inactive uveitis remission. **(B)** Forest plot of studies reporting the remission rate of ocular inflammation.

Sustained remission was defined as the complete absence of disease activity maintained for at least 3 consecutive months during follow-up. This outcome was reported in nine studies involving 324 patients. The pooled sustained remission rate was 57.08% (95% CI: 46.94%–66.96%; I^2^ = 60.79%; Figure 2A).

#### 3.4.2 Ocular inflammation resolution

Ocular inflammation resolution was defined as a ≥2-step decrease or complete resolution (grade 0) in anterior chamber cells or vitreous haze according to the SUN Working Group criteria. Twelve studies comprising a total of 342 patients reported this outcome following tocilizumab therapy. Among them, 242 patients achieved resolution. As shown in Figure 2B, the pooled proportion of patients achieving ocular inflammation resolution was 75.23% (95% CI: 64.04%–85.09%; I^2^ = 72.77%).

#### 3.4.3 ME resolution and CMT reduction

**FIGURE 3:**
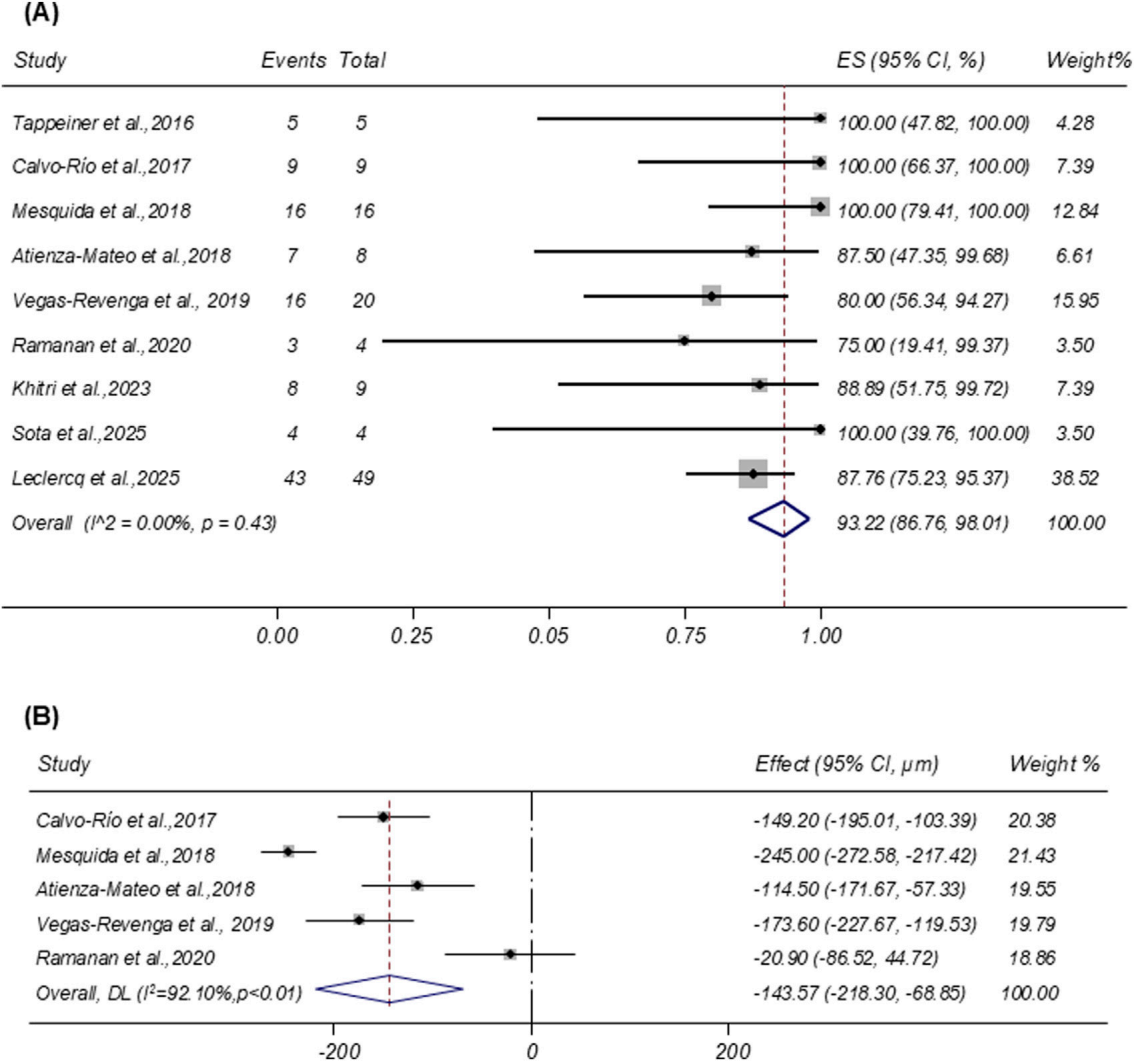
**(A)** Forest plot of studies reporting the remission rate of macular edema. **(B)** Forest plot of studies reporting the central macular thickness reduction (µm).

Nine studies reported the resolution of ME after tocilizumab therapy. Pooled analysis showed a remission rate of 93.22% (95% CI: 86.76%–98.01%; I^2^ = 0.00%; Figure 3A). Five studies provided CMT as mean ± SD. Random-effects pooling demonstrated a mean CMT reduction of −143.57 µm (95% CI: −218.30 to −68.85 µm; I^2^ = 92.10%; Figure 3B).

#### 3.4.4 VA improvement

**FIGURE 4:**
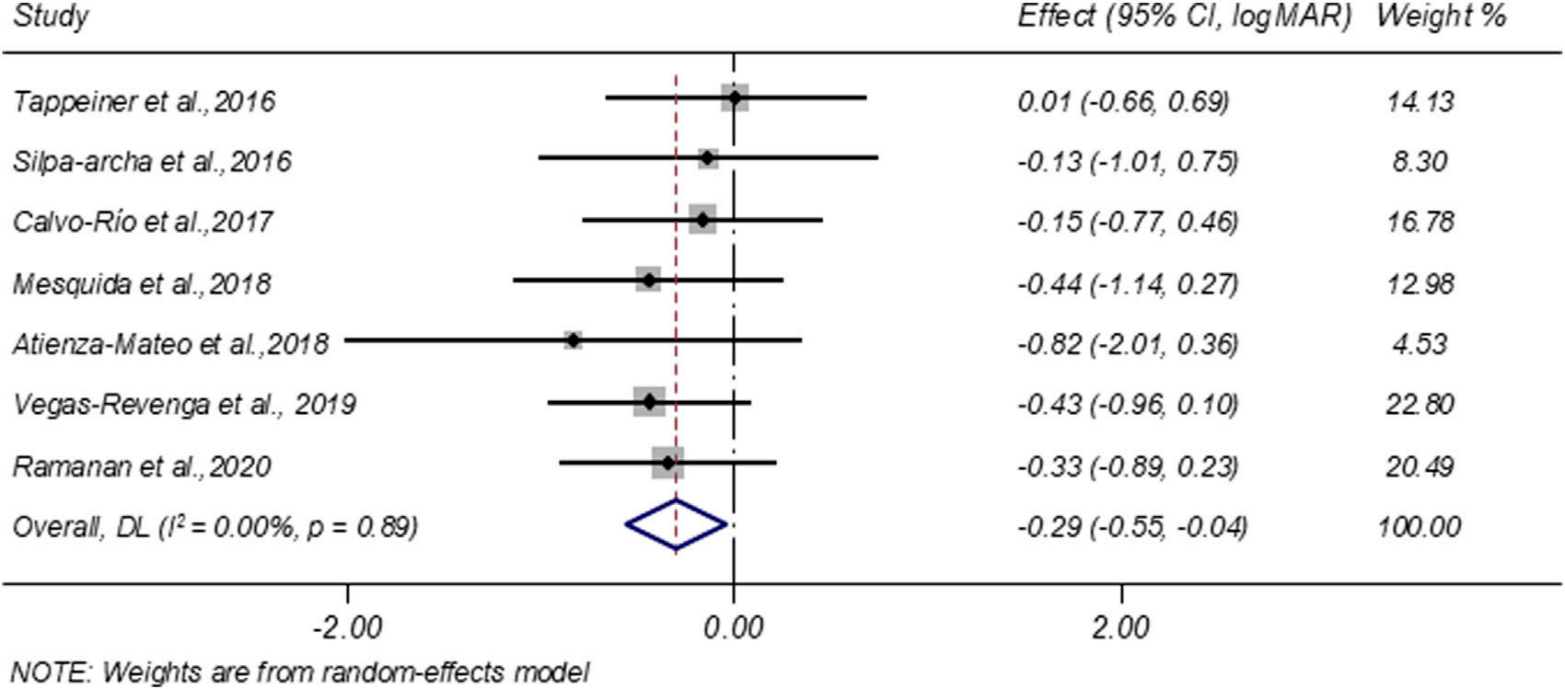
Forest plot of studies reporting the improvement of vision acuity (logMAR).

Seven studies evaluated VA before and after treatment. A random-effects meta-analysis demonstrated a significant improvement in VA following tocilizumab therapy, with a pooled change of −0.29 LogMAR (95% CI: −0.55 to −0.04; I^2^ = 0.00%; Figure 4).

#### 3.4.5 Glucocorticoid tapering and discontinuation

**FIGURE 5:**
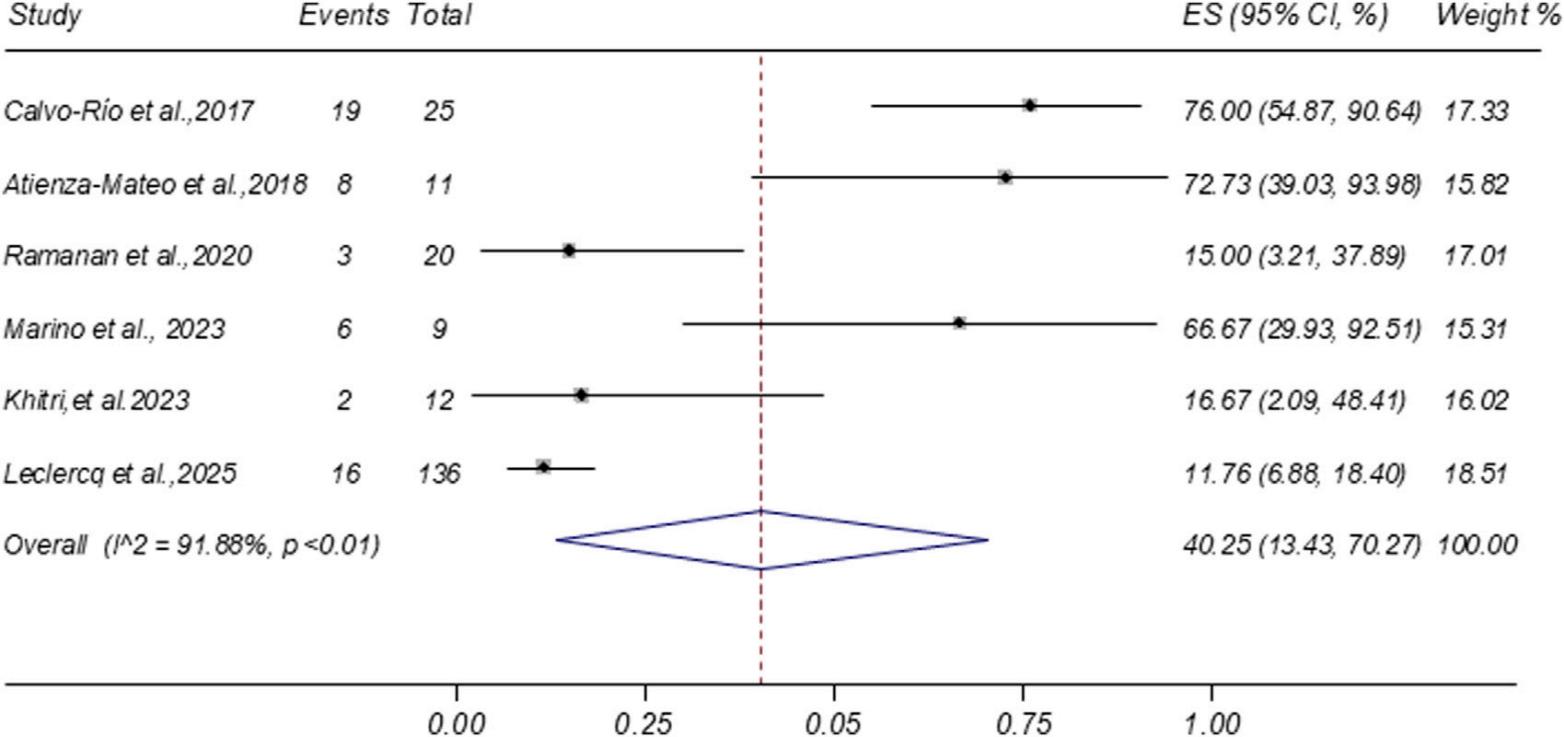
Forest plot of studies reporting the rate of glucocorticoid discontinuation.

Six studies reported data on glucocorticoid discontinuation rates following tocilizumab treatment. The meta-analysis yielded a pooled discontinuation rate of 40.25% (95% CI: 13.43%–70.27%; I^2^ = 91.88%), as shown in Figure 5. Two additional studies reported the proportion of patients achieving at least a 50% reduction in glucocorticoid dosage (pooled proportion 56.58%; 95% CI: 38.59%–73.80%), as presented in Supplementary Figure S1. Furthermore, a separate study noted that five out of seven patients (71.43%) were receiving ≤5 mg/day of systemic corticosteroids at the final follow-up visit (Silpa-archa et al., 2016).

#### 3.4.6 AEs and SAEs

**FIGURE 6:**
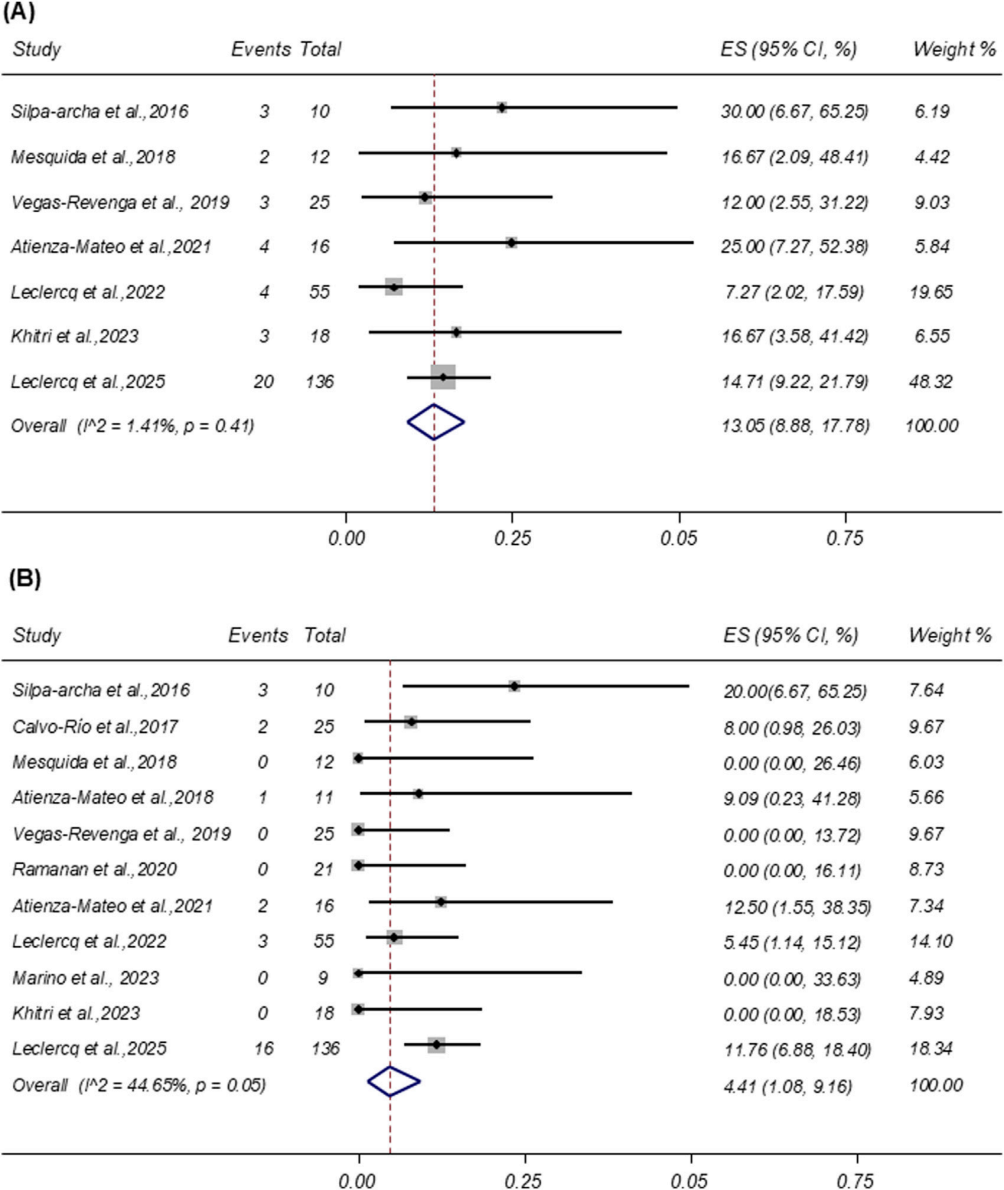
**(A)** Forest plot of studies reporting the rate of adverse events. **(B)** Forest plot of studies reporting the rate of severe adverse events. LogMAR = logarithm of the minimum angle of resolution; ES, effect size; CI, confidence interval; DL, DerSimonian–Laird random-effects model.

AEs were reported in seven studies involving 272 patients. As shown in Figure 6A, the pooled incidence of AEs was 13.05% (95% CI: 8.88%–17.78%), with low heterogeneity (I^2^ = 1.41%; p = 0.41). The most frequently reported AEs were infections, accounting for 46.15% (n = 18) of all events. SAEs were reported in 11 studies involving 338 patients. The pooled incidence of SAEs was 4.41% (95% CI: 1.08%–9.16%; I^2^ = 44.65%), based on a random-effects model (Figure 6B). Similar to AEs, infections were the most common SAEs, representing 37.04% (n = 10) of all reported serious events.

#### 3.4.7 Sensitivity analysis

To evaluate the robustness of the meta-analytic findings, leave-one-out sensitivity analyses were performed. As illustrated in Supplementary Figure S2, the overall results remained stable after sequential exclusion of individual studies, confirming the consistency and reliability of the pooled estimates.

#### 3.4.8 Subgroup analysis

To explore the sources of heterogeneity observed in the primary outcomes, prespecified subgroup analyses were conducted based on disease etiology (JIA vs. BD), patient age (<30 years vs. ≥ 30 years), treatment duration (<9 months vs. ≥ 9 months), and follow-up length (<18 months vs. ≥ 18 months) whenever I^2^ exceeded 25% (Table 2). A 30-year age cutoff was applied, corresponding approximately to the median age of the pooled study populations. Heterogeneity was classified as low (I^2^ ≤ 25%), moderate (25% < I^2^ ≤ 50%), substantial (50% < I^2^ ≤ 75%), and considerable (I^2^ > 75%).

**TABLE 2:** Subgroup analyses of ocular outcomes and safety among patients with NIU treated with tocilizumab.

Subgroup	Number of studies	Pooled ES (%)	95% CI (%)	I^2^ (%)	p-value
Ocular inflammation sustained remission (≥3 months)	9	57.08	46.94–66.96	60.79	<0.01
Treatment duration					
–Average duration <9 months	5	53.22	37.91–68.24	77.99	<0.01
–Average duration ≥9 months	4	62.97	50.86–74.39	0.00	0.82
Age-group					
–Average age of <30 years	4	59.15	39.78–77.27	66.14	0.03
–Average age of ≥30 years	5	55.59	42.65–68.18	64.29	0.02
Disease etiology					
–JIA	3	56.63	30.73–80.87	NA	NA
–BD	3	66.75	51.70–80.40	NA	NA
Follow-up duration					
–Average <18 months	5	59.10	43.29–74.08	57.05	0.05
–Average ≥18 months	4	55.32	40.14–70.04	72.33	0.01
Ocular inflammation resolution	12	75.23	64.04–85.09	72.77	<0.01
Treatment duration					
–Average duration <9 months	5	64.55	47.78–79.76	80.49	<0.01
–Average duration ≥9 months	7	83.33	58.58–96.42	34.40	0.17
Age-group					
–Average age of <30 years	5	67.08	61.64–88.50	80.18	<0.01
–Average age of ≥30 years	7	79.75	67.62–89.87	67.67	<0.01
Disease etiology					
–JIA	4	62.36	31.72–88.83	82.36	0.06
–BD	3	89.34	73.55–99.12	NA	NA
Follow-up duration					
–Average <18 months	6	78.62	53.09–96.64	84.29	<0.01
–Average ≥18 months	6	71.33	62.39–79.57	33.03	0.19
Glucocorticoid discontinuation	6	40.25	13.43–70.27	91.88	<0.01
Treatment duration					
–Average duration <9 months	3	31.62	1.87–73.94	NA	NA
–Average duration ≥9 months	3	50.83	15.42–85.83	NA	NA
Age-group					
–Average age of <30 years	4	42.42	10.67–77.70	87.34	<0.01
–Average age of ≥30 years	2	13.88	8.31–20.41	NA	NA
Disease etiology					
–JIA	3	51.45	11.45–90.46	NA	NA
–BD	2	42.42	22.16–63.91	NA	NA
Follow-up duration					
–Average duration <18 months	3	53.68	13.29–91.58	NA	NA
–Average duration ≥18 months	3	26.31	2.88–59.46	NA	NA
Serious adverse events	11	4.41	1.08–9.16	44.65	0.05
Treatment duration					
–Average duration <9 months	4	6.60	2.27–12.56	43.58	0.15
–Average duration ≥9 months	7	3.37	0.00–11.71	47.60	0.08
Age-group					
–Average age of <30 years	4	1.15	0.00–6.33	0.00	0.46
–Average age of ≥30 years	7	6.56	1.76–13.28	49.35	0.07
Disease etiology					
–JIA	3	1.85	0.00–8.94	NA	NA
–BD	3	4.95	0.00–17.57	NA	NA
Follow-up duration					
–Average duration <18 months	5	4.78	0.00–15.90	60.44	0.04
–Average duration ≥18 months	6	5.15	1.34–10.51	30.53	0.21

NIU, noninfectious uveitis; NA, not applicable due to the limited number of studies; ES, effect size; CI, confidence interval; JIA, juvenile idiopathic arthritis; BD, Behçet’s disease.

Subgroup analyses were conducted using random-effects models. Heterogeneity was quantified using the I^2^ statistic and Cochran’s Q test; p < 0.05 was interpreted as substantial heterogeneity. Heterogeneity was classified as low (I^2^ ≤ 25%), moderate (25% < I^2^ ≤ 50%), substantial (50% < I^2^ ≤ 75%), and considerable (I^2^ > 75%), and I^2^ values ≤ 25% are in bold.

Patients receiving tocilizumab for ≥9 months demonstrated higher rates of sustained ocular inflammation remission (62.97% vs. 53.22%), with heterogeneity disappearing in the longer-duration subgroup (I^2^ = 0.00%; p = 0.82). This finding should be interpreted with caution because it was derived from only four studies in the ≥9-month treatment-duration subgroup. Similarly, the meta-analysis of overall ocular inflammation resolution showed substantial heterogeneity (I^2^ = 72.77%; p < 0.0.1). However, subgroup analysis based on treatment duration revealed moderate heterogeneity in the ≥9-month group (I^2^ = 34.40%; p = 0.17), along with a higher resolution rate than that in the <9-month group (83.33% vs. 64.55%). Regarding SAEs, pooled analysis showed moderate heterogeneity (I^2^ = 44.65%; p = 0.05). Subgroup analyses revealed lower heterogeneity in studies with patients aged <30 years (I^2^ = 0.00%; p = 0.46) and with follow-up durations ≥18 months (I^2^ = 30.53%; p = 0.21). However, this observation should also be interpreted cautiously given the limited number of studies (n = 4) in the <30-year subgroup. Taken together, these findings indicate that between-study heterogeneity could be attributed to differences in patient characteristics and study design.

#### 3.4.9 Publication bias

Publication bias was assessed using Egger’s test, and no significant bias was detected across any of the pooled analyses (p > 0.05 for all outcomes; Supplementary Table S4).

## 4 Discussion

In this study, we provide the first quantitative synthesis evaluating the efficacy and safety of tocilizumab in refractory NIU associated with autoimmune and inflammatory diseases. The findings suggest that tocilizumab provides meaningful clinical benefits, including inflammation remission, ME resolution, VA improvement, and glucocorticoid-sparing effects, with an acceptable safety profile.

When compared with existing anti-TNF therapies, our pooled estimates for inflammation control (75%) demonstrate comparable efficacy. For example, adalimumab has shown activity control rates of 74% at ≤6 months and 79% at ≥12 months in NIU (Ming et al., 2018), whereas a 2020 meta-analysis focusing on BD-associated uveitis reported an inflammation resolution rate of 68% (Hu et al., 2020). These results support IL-6 receptor blockade as a promising second-line biologic for patients unresponsive to conventional immunosuppressants and anti-TNF therapies.

ME is a major contributor to vision loss in NIU, and its resolution remains a critical therapeutic goal. In our meta-analysis, tocilizumab achieved a pooled ME resolution rate of 93.22%, with no significant heterogeneity across studies (I^2^ = 0.00%; p = 0.43), indicating a consistent treatment effect. Furthermore, treatment was associated with a mean reduction in CMT of 143.57 µm, highlighting the anatomical improvements achieved through IL-6 inhibition.

A retrospective study from the French Uveitis Network compared the efficacy of anti-TNF-α agents and tocilizumab in refractory ME secondary to NIU. The findings demonstrated that tocilizumab doubled the odds of achieving complete resolution of uveitic ME compared with anti-TNF-α therapies (Leclercq et al., 2022). The superior efficacy of tocilizumab likely reflects the pivotal role of IL-6 in promoting vascular permeability and disrupting the blood–retinal barrier, which are the key pathophysiological mechanisms in the development of uveitic ME (Yang et al., 2023). IL-6 acts as a pleiotropic cytokine that exacerbates intraocular inflammation through Th17 polarization, B-cell activation, and VEGF upregulation, collectively driving blood–retinal barrier breakdown and increased vascular permeability. Therefore, IL-6 inhibition directly targets these pathogenic pathways, providing a mechanistically rational approach for resolving macular thickening (Chen et al., 2011; Mesquida et al., 2014; McElvaney et al., 2021; Aliyu et al., 2022). Notably, two phase III trials, namely, MEERKAT (NCT05642312) and SANDCAT (NCT05642325), are currently underway to evaluate the efficacy, safety, pharmacokinetics, and pharmacodynamics of vamikibart, an anti-IL-6 monoclonal antibody, in patients with uveitis ME (Suhler et al., 2024). Furthermore, in a phase II study, sarilumab, an anti-IL-6 receptor monoclonal antibody, demonstrated clinical benefits in NIU involving the posterior segment, particularly in patients with ME (Heissigerová et al., 2019). These ongoing studies further support the potential use of IL-6-targeted therapies for patients with uveitis complicated by persistent or vision-threatening ME.

VA serves as a direct and patient-centered indicator of treatment efficacy in uveitis. In this meta-analysis, tocilizumab therapy led to a mean improvement of −0.29 logMAR, equivalent to a gain of approximately 14 ETDRS letters, or nearly three lines on a Snellen chart, a threshold widely accepted as clinically meaningful (Anothaisintawee et al., 2012). Importantly, no statistical heterogeneity was detected across the included studies (I^2^ = 0.00%; p = 0.89), suggesting robust consistency in the visual benefit. The notable VA improvement associated with tocilizumab may be explained by its dual mechanism—simultaneously reducing intraocular inflammation and resolving ME—both of which critically impact visual outcomes (Hassan et al., 2019). These findings highlight the clinical value of tocilizumab, particularly in patients with chronic uveitis complicated by ME.

Reducing systemic glucocorticoid exposure remains a critical objective in the long-term NIU management, given the risk of complications, such as cataract, glaucoma, osteoporosis, and metabolic disorders (Hassan et al., 2019). In this meta-analysis, tocilizumab demonstrated a substantial glucocorticoid-sparing effect, with a pooled glucocorticoid discontinuation rate of 40.25% at the final follow-up. By comparison, a meta-analysis of adalimumab in NIU reported corticosteroid sparing in 82.0% of patients, with 48.8% able to completely discontinue corticosteroids (Ming et al., 2018). Although adalimumab showed a higher corticosteroid-sparing rate, direct comparisons are limited by differences in study design, patient populations, and baseline disease severity. Nonetheless, the corticosteroid discontinuation rate observed with tocilizumab remains clinically meaningful, particularly in refractory cases.

Although tocilizumab effectively reduces disease activity and improves anatomical and visual outcomes, its safety profile remains a key consideration. The pooled incidence of AEs and SAEs was 17.46%, consistent with rates reported in meta-analyses of tocilizumab use in systemic JIA and rheumatoid arthritis (Aeschlimann et al., 2020; Cordero-Alfaro et al., 2021; Specker et al., 2021). Furthermore, in rheumatoid arthritis patients refractory to anti-TNF agents, tocilizumab demonstrated efficacy and SAEs comparable to those of rituximab (Humby et al., 2021). Additionally, transient, dose-dependent neutropenia has been reported with tocilizumab therapy (Papo et al., 2014; Shovman et al., 2015). Taken together, these findings support tocilizumab as a viable therapeutic option for refractory NIU, with a potentially favorable benefit–risk balance when infection screening and routine hematologic monitoring are incorporated into clinical practice.

The methodological quality of the included studies was generally moderate to high based on the NOS and JBI assessments. However, the predominance of retrospective, single-arm designs, together with variability in sample size, outcome reporting, and follow-up duration, may introduce bias and limit the generalizability of the pooled findings.

This study has several limitations. First, the majority of included studies (10 of 13) were retrospective, single-arm case series, reflecting the later adoption of tocilizumab relative to other biologics in NIU. We addressed potential biases through rigorous quality assessment (NOS and JBI tools) and sensitivity analyses. Second, heterogeneity in baseline characteristics, such as disease duration prior to tocilizumab initiation and patient demographics, may have influenced outcomes. Likewise, variable reporting of concomitant therapies, including systemic corticosteroids and conventional immunosuppressants, may confound the independent effects attributed to tocilizumab. Third, subgroup findings by age should be interpreted with caution. The 30-year cutoff was determined based on the median age distribution to balance subgroup sample sizes. Because only a few studies focused exclusively on pediatric patients and several included overlapping age ranges, an 18-year threshold analysis was not feasible. Future studies that report outcomes separately for pediatric and adult patients will enable more robust and clinically meaningful comparisons. Finally, our analysis also included several studies that enrolled patients with ocular-limited, nonsystemic inflammatory uveitis, such as idiopathic or Birdshot chorioretinopathy. Future prospective studies should stratify outcomes for systemic versus ocular-limited NIU to better define the scope of tocilizumab efficacy.

Despite these limitations, this meta-analysis provides valuable and comprehensive insights into the efficacy and safety of tocilizumab in refractory NIU. The evidence supports its role as a second-line biologic agent, particularly for patients with chronic or vision-threatening ME. Given its favorable benefit–risk profile, earlier use of tocilizumab may be considered in selected cases, such as refractory ME with controlled inflammation. Although concomitant immunosuppressants may introduce confounding effects, certain combination regimens could yield synergistic therapeutic benefits, warranting evaluation in future prospective studies. Accordingly, vigilant monitoring for infectious complications remains essential, particularly during early treatment phases or when combined with other immunosuppressive agents.

## 5 Conclusion

In this systematic review and meta-analysis, we demonstrate that tocilizumab offers meaningful clinical benefits in refractory NIU associated with systemic autoimmune and inflammatory diseases, achieving substantial inflammation control, resolution of ME, improvement in VA, and a corticosteroid-sparing effect, with a relatively low incidence of adverse events. These findings support IL-6 receptor blockade as a viable second-line option in patients unresponsive to conventional immunosuppressants and anti-TNF-α agents. Further large-scale, high-quality randomized controlled trials are warranted to confirm these results and define optimal treatment regimens.

## Data Availability

The original contributions presented in the study are included in the article/Supplementary Material; further inquiries can be directed to the corresponding author.

## Abbreviations

AEs, adverse events; anti-TNF-α, antitumor necrosis factor alpha; BD, Behçet’s disease; CMT, central macular thickness; IL-6, interleukin-6; JBI, Joanna Briggs Institute; JIA, juvenile idiopathic arthritis; logMAR, logarithm of the minimum angle of resolution; MeSH, Medical Subject Headings; ME, macular edema; NIU, refractory noninfectious uveitis; NOS, Newcastle–Ottawa Scale; PRISMA, Preferred Reporting Items for Systematic Reviews and Meta-Analyses; SAEs, serious adverse events; SUN, Standardization of Uveitis Nomenclature; VA, visual acuity; VEGF, vascular endothelial growth factor.
