# Supplementary figures and images for: Effectiveness and safety of tocilizumab in refractory noninfectious uveitis: a systematic review and meta-analysis
Source: Front Pharmacol. 2025 Dec 4;16:1694311. doi: 10.3389/fphar.2025.1694311 (PMC12711790; doi:10.3389/fphar.2025.1694311)

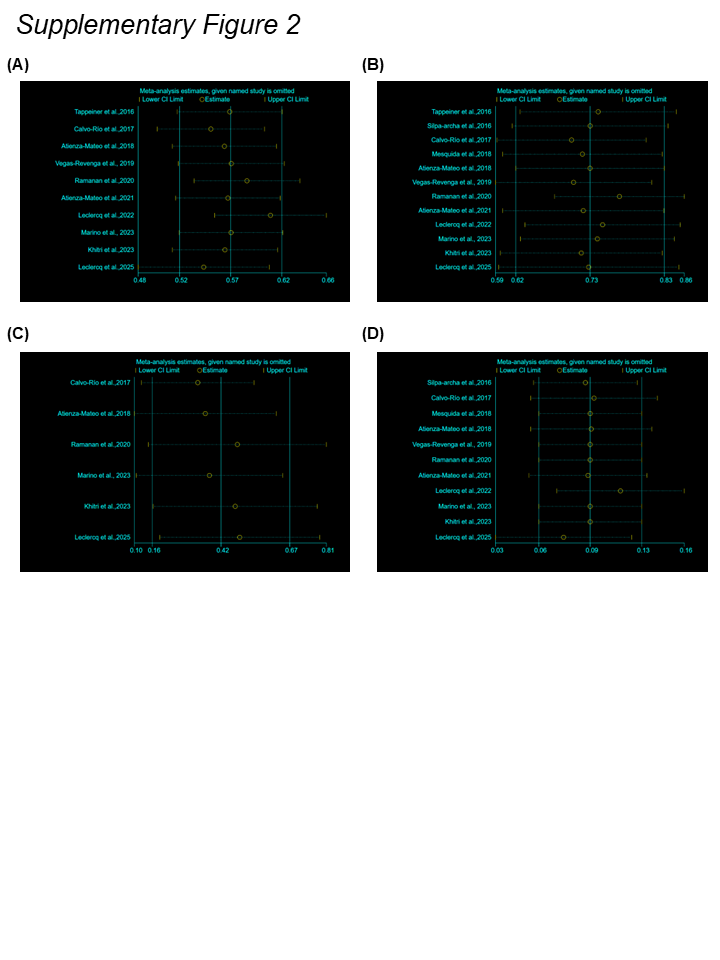

Supplement: Supplementary file 2 [file Image2.tif]

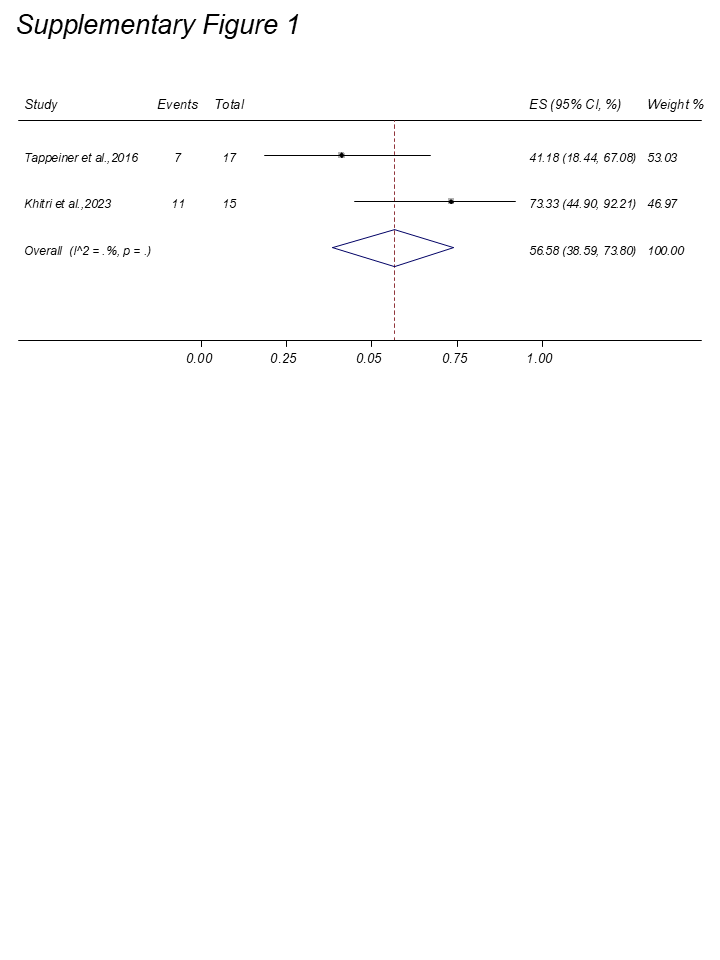

Supplement: Supplementary file 3 [file Image1.tif]
