# Supplementary material for: Effectiveness and safety of tocilizumab in refractory noninfectious uveitis: a systematic review and meta-analysis
Source: Front Pharmacol. 2025 Dec 4;16:1694311. doi: 10.3389/fphar.2025.1694311 (PMC12711790; doi:10.3389/fphar.2025.1694311)
Supplement: Supplementary file 5 [file Table3.docx]

Supplementary Material

# Supplementary Tables

**Supplementary Table 1.** Searching strategy

| **Database** | **Searching Strategy** |
| --- | --- |
| PubMed | 1. ((((((((((((((((((Interleukin-6 Inhibitors[MeSH Terms]) ) OR (Inhibitors, Interleukin-6[Title/Abstract])) OR (IL-6 Inhibitors[Title/Abstract])) OR (IL 6 Inhibitors[Title/Abstract])) OR (Anti-IL-6 Agents[Title/Abstract])) OR (Agents, Anti-IL-6[Title/Abstract])) OR (Anti IL 6 Agents[Title/Abstract])) OR (IL6 inhibitors[Title/Abstract])) OR (inhibitors, IL6[Title/Abstract])) OR (Anti-IL6 Agents[Title/Abstract])) OR (Agents, Anti-IL6[Title/Abstract])) OR (Anti IL6 Agents[Title/Abstract])) OR (Anti-Interleukin-6 Agents[Title/Abstract])) OR (Agents, Anti-Interleukin-6[Title/Abstract])) OR (Tocilizumab[Title/Abstract])) OR (Sarilumab[Title/Abstract]) OR (Siltuximab[Title/Abstract])OR (Olokizumab[Title/Abstract]) 2. (Uveitis [MeSH Terms]) OR (Uveitides[Title/Abstract]) 3. 1 and 2 |
| Web of Science | 1. (((((((((((((((TS=(Interleukin-6 Inhibitors)) OR TS=(Inhibitors, Interleukin-6)) OR TS=(IL-6 Inhibitors)) OR TS=(IL 6 Inhibitors)) OR TS=(Anti-IL-6 Agents)) OR TS=(Agents, Anti-IL-6)) OR TS=(Anti IL 6 Agents)) OR TS=(IL6 inhibitors)) OR TS=(inhibitors, IL6)) OR TS=(Anti-IL6 Agents)) OR TS=(Agents, Anti-IL6)) OR TS=(Anti IL6 Agents)) OR TS=(Anti-Interleukin-6 Agents)) OR TS=(Agents, Anti-Interleukin-6)) OR TS=(Tocilizumab)) OR TS=(Sarilumab) 2. (TS=(Uveitis)) OR TS=(Uveitides) 3. 1 and 2 |
| Embase | 1. 'interleukin 6 inhibitor'/exp OR 'interleukin 6 inhibitor':ti,ab OR 'il-6 inhibitor':ti,ab OR 'il 6 inhibitor':ti,ab OR 'anti-il-6 agent':ti,ab OR 'anti il 6 agent':ti,ab OR 'anti-il6 agent':ti,ab OR 'anti il6 agent':ti,ab OR 'anti-interleukin 6 agent':ti,ab OR 'anti interleukin 6 agent':ti,ab OR tocilizumab:ti,ab OR sarilumab:ti,ab OR siltuximab:ti,ab OR olokizumab:ti,ab  2. 'uveitis'/exp OR uveitis:ti,ab OR uveitides:ti,ab  3. 1 AND 2 |
| Cochrane Library | #1 MeSH descriptor: [Interleukin-6] explode all trees  #2 MeSH descriptor: [Interleukin-6 Antagonists] explode all trees  #3 tocilizumab:ti,ab,kw  #4 sarilumab:ti,ab,kw  #5 siltuximab:ti,ab,kw  #6 olokizumab:ti,ab,kw  #7 "IL-6 inhibitor":ti,ab,kw OR "IL 6 inhibitor":ti,ab,kw OR "Anti-IL6":ti,ab,kw OR "Anti IL6":ti,ab,kw OR "Anti-interleukin-6":ti,ab,kw OR "Interleukin-6 blocking":ti,ab,kw  #8 #1 OR #2 OR #3 OR #4 OR #5 OR #6 OR #7  #9 MeSH descriptor: [Uveitis] explode all trees  #10 uveitis:ti,ab,kw OR uveitides:ti,ab,kw  #11 #9 OR #10  #12 #8 AND #11 |
| ClinicalTrials | (tocilizumab OR sarilumab OR siltuximab OR olokizumab OR "IL-6 inhibitor" OR "interleukin-6 inhibitor" OR "anti-IL6" OR "anti-IL-6") AND (uveitis OR uveitides) |

**Supplementary Table 2.**  JBI Critical Appraisal Checklist for Case Series for included retrospective studies

| Study | Q1 | Q2 | Q3 | Q4 | Q5 | Q6 | Q7 | Q8 | Q9 | Q10 | Total |
| --- | --- | --- | --- | --- | --- | --- | --- | --- | --- | --- | --- |
| Tappeiner et al.,2016 | ✓ | ✓ | ✓ | ✓ | ✓ | ✓ | ✓ | ✕ | ✓ | ✓ | 9 |
| Silpa-archa et al.,2016 | ✓ | ✓ | ✓ | ✓ | ✓ | ✓ | ✓ | ✓ | ✓ | ✓ | 10 |
| Calvo-Río et al.,2017 | ✓ | ✓ | ✓ | ✓ | ✓ | ✓ | ✓ | ✓ | ✓ | ✓ | 10 |
| Mesquida et al.,2018 | ✓ | ✓ | ✓ | ✓ | ✓ | ✓ | ✕ | ✓ | ✓ | ✓ | 9 |
| Atienza-Mateo et al.,2018 | ✓ | ✓ | ✓ | ✓ | ✓ | ✓ | ✓ | ✓ | ✓ | ✓ | 10 |
| Vegas-Revenga et al., 2019 | ✓ | ✓ | ✓ | ✓ | ✓ | ✓ | ✓ | ✓ | ✓ | ✓ | 10 |
| Ramanan et al.,2020 | ✓ | ✓ | ✓ | ✓ | ✓ | ✓ | ✕ | ✕ | ✓ | ✓ | 8 |
| Atienza-Mateo et al.,2021 | ✓ | ✓ | ✓ | ✓ | ✓ | ✓ | ✓ | ✓ | ✓ | ✓ | 10 |
| Marino et al., 2023 | ✓ | ✓ | ✓ | ✓ | ✓ | ✓ | ✓ | ✓ | ✓ | ✓ | 10 |
| Khitri et al.,2023 | ✓ | ✓ | ✓ | ✓ | ✓ | ✓ | ✕ | ✓ | ✓ | ✓ | 9 |
| Sota et al.,2025 | ✓ | ✓ | ✓ | ✓ | ✓ | ✓ | ✕ | ✕ | ✓ | ✓ | 8 |

JBI = Joanna Briggs Institute; NIU = non-infectious uveitis.

The JBI Critical Appraisal Checklist for Case Series contains 10 items:

Q1 – Were there clear criteria for inclusion in the case series?

Q2 – Was the condition measured in a standard, reliable way for all participants included in the case series?

Q3 – Were valid methods used for identification of the condition for all participants included in the case series?

Q4 – Did the case series have consecutive inclusion of participants?

Q5 – Did the case series have complete inclusion of participants?

Q6 – Was there clear reporting of the demographics of the participants in the study?

Q7 – Was there clear reporting of the clinical information of the participants?

Q8 – Were the outcomes or follow-up results of cases clearly reported?

Q9 – Was there clear reporting of the presenting site(s)/clinic(s) demographic information?

Q10 – Was statistical analysis appropriate?

Each item is scored ✓ (Yes) when fully met, ✕ (No/Unclear) when not met or not reported. The total score (0–10) in the right-most column reflects overall methodological quality ( ≥8 = high, 5–7 = moderate, ≤ 4 = low).

**Supplementary Table 3.**  Quality assessment using Newcastle-Ottawa Scale

| STUDY | SELECTION (max 4-points) | | | | COMPARABILITY (max 2-points) | OUTCOME (max 3-points) | | | OVERALL (out of 9) |
| --- | --- | --- | --- | --- | --- | --- | --- | --- | --- |
|  | Representativeness  of  the tocilizumab group | Selection  of the  control group | Ascertainment of  tocilizumab group | Demonstration  that outcome of  interest was not present at start of study | Comparability of  cohorts based on  the design or analysis | Assessment  of outcome | Was  follow-up  long  enough for  outcomes  to occur? | Adequacy of the follow-up |  |
| Leclercq et al.,2022 | 1 | 1 | 1 | 1 | 2 | 1 | 1 | 0 | 8 |
| Leclercq et al.,2025 | 1 | 1 | 1 | 1 | 1 | 1 | 1 | 0 | 7 |

“1” denotes the study met the item and was awarded one star; “2” (only possible for Comparability) indicates two stars; “0” indicates the criterion is not met (no star). Overall score of 6 to 7 considered as moderate quality and 8 to 9 as high quality.

**Supplementary Table 4.**  Publication bias

| **Outcome** | **Egger's Test (p-value)** | **Evidence of Bias** |
| --- | --- | --- |
| **Ocular inflammation sustained remission** | 0.86 | NO |
| **Ocular inflammation resolution** | 0.46 | NO |
| **Macular edema resolution** | 0.34 | NO |
| **Visual acuity improvement** | 0.66 | NO |
| **Glucocorticoids discontinuation** | 0.10 | NO |
| **Adverse events** | 0.17 | NO |
| **Serious adverse events** | 0.38 | NO |

# Supplementary Figures legends

**Supplementary Figure 1.**

Forest plot of the proportion of patients achieving a ≥50% reduction in systemic glucocorticoids dose.

**Supplementary Figure 2.**

Sensitivity analysis. (A) Sensitivity analysis for ocular inflammation sustained remission; (B) Sensitivity analysis for ocular inflammation resolution; (C) Sensitivity analysis for glucocorticoids discontinuation; (D) Sensitivity analysis for serious adverse events.
